# Synergistic Therapeutic Effects of Low Dose Decitabine and NY-ESO-1 Specific TCR-T Cells for the Colorectal Cancer With Microsatellite Stability

**DOI:** 10.3389/fonc.2022.895103

**Published:** 2022-06-14

**Authors:** Ganjun Yu, Wenying Wang, Xiaobo He, Jia Xu, Rongrong Xu, Tao Wan, Yanfeng Wu

**Affiliations:** Department of Immunology, College of Basic Medicine & National Key Laboratory of Medical Immunology, Naval Medical University, Shanghai, China

**Keywords:** decitabine, T cell receptor engineered T cell, synergistic therapy, colorectal cancer, microsatellite stability

## Abstract

Patients of colorectal cancer (CRC) with microsatellite stability (MSS) show poor clinical response and little beneficial result from the immune-checkpoint inhibitors, due to the ‘cold’ tumor microenvironment. Meanwhile, decitabine can drive the ‘cold’ microenvironment towards ‘hot’ in multiple ways, such as upregulating the tumor associated antigen (TAA) and human leukocyte antigen (HLA) molecular. NY-ESO-1, one of the most important TAAs, can be observably induced in tumors by low dose decitabine, and present itself as ideal targets for antigen specific T cell receptor engineered T (TCR-T) cells. We innovatively used a synergistic tactic, combining decitabine and NY-ESO-1 specific TCR-T cells, for fighting the MSS CRC. Firstly, we confirmed the lysing effect of the NY-ESO-1 TCR-T cells on the NY-ESO-1^+^ and HLA-A2^+^ cells *in vitro* and *in vivo*. In A375 tumor-bearing mice, the results showed that NY-ESO-1 TCR-T cell therapy could inhibit A375 tumor growth and prolonged the survival time. Furthermore, the synergistic effect of decitabine and NY-ESO-1 TCR-T cells was shown to induce an even higher percentage of tumor cells being lysed *in vitro* than other control groups, and more potent tumor inhibition and longer survival time were observed *in vivo*. The innovative synergistic therapeutic strategy of decitabine and TCR-T cells for the CRC with MSS may be also effective in the treatment of other epithelial malignancies. Decitabine may likewise be adopted in combination with other cellular immunotherapies.

## Introduction

Colorectal cancer (CRC) is a major cause of cancer-related death worldwide. In developed countries, 25-50% of the patients are diagnosed at early stages, but still go on to develop into metastatic disease and approximately 25% when diagnosed are already present with advanced stage disease (1–5). Although immunotherapy has dramatically changed the landscape of treatment for many advanced cancers, the benefit in CRC has thus far been limited to patients with microsatellite stability (MSS).

Microsatellites are repeated sequences of DNA. The DNA is considered as stable when the number of microsatellite repeats is the same in all cells of the body, also referred to as microsatellite stable or MSS. MSI refers to any change in microsatellite length caused by insertion or deletion of a repeat unit in a microsatellite in a tumor, as opposed to normal tissues, which is the condition of genetic hypermutability as a result of deficient MMR (6). The presence of MSI is the phenotypic evidence that MMR is not functioning normally. PD-1 blockades have been approved by the Food and Drug Administration (FDA) for the treatment of solid tumors with microsatellite instability-high (MSI-H) or mismatch repair deficient (dMMR), including colorectal cancer (CRC). However, the checkpoint inhibitors’ are unable to act in front of the MSS CRC (7–9).

Unfortunately, there are only approximately 10-20% with the MSI-H characteristics in all CRC patients (6, 10, 11). The majority of the remainder are microsatellite instability-low (MSI-L) or MSS (12). For these patients, the key approach to getting benefit from the immunotherapy is turning their tumors from the ‘cold’ into the ‘hot’ state (13, 14). Studies have shown that low dose decitabine (DAC) can not only significantly induce and increase the expression of tumor antigens, such as NY-ESO-1, MAGE-A3/6, but also multiply the expression of immune markers such as CD80, MHC-I molecules, and then improve the infiltration of immune cells into tumor sites, providing a more appropriate immune microenvironment for immune checkpoint inhibitors, therapeutic vaccines, adoptive cell therapy, and other immunotherapies (15–21).

Because T cells are the final effectors of immune-mediated cancer regression, strategies that direct tumor-specific T cells infusion have been developed and have produced impressive results in hematological malignancies and some solid cancers (22, 23). Clinical successes of T cell receptor-engineered T (TCR-T) cell therapy were achieved in malignant melanoma (24, 25), and subsequently in synovial sarcoma (24, 25), and multiple myeloma (26), non-small cell lung cancer (27), neuroblastoma (28), specifical with a TCR against the cancer testis antigen NY- ESO-1, with objective response rates of 45-67%. In 2016, NY-ESO-1 specific TCR-T cell therapy developed by Adaptimmune Therapeutics PLC was granted orphan drug status by U.S. Food and Drug Administration (FDA), which reaffirmed the recognition by regulators and increased the expectation of the immunotherapy of solid tumors.

Therefore, we artificially constructed NY-ESO-1 specific TCR-T cells, innovatively combined with DAC together for those CRC with MSS. The results we reported here indicated that DAC could induce and improve the NY-ESO-1 expression in CRC cells, especially in MSS cell lines, which could be more effectively recognized and destroyed by the adoptively transferred TCR-T cells. Here we showed a potential therapeutic strategy with DAC and TCR-T cells against MSS CRC.

## Materials and Methods

### Animals, Peripheral Blood Lymphocytes (PBLs), and Cell Lines

The BALB/c nude mice were obtained from Shanghai Sippr-BK laboratory animal Co. Ltd. The NOD-Prkdc^scid^ Il2rg^null^ (NPG) mice were obtained from Beijing Vitalstar Biotechnology Co., Ltd. All mice were 6- to 8-week-old males bred and maintained in specific pathogen-free conditions (Institutional Animal Care and Use Committee number: SYXK[Shanghai]2015-0028).

All the PBLs involved in this study were obtained from healthy donors at Changhai Hospital, Shanghai, China, with the approval of the local ethics committee. The human CRC cell lines HCT116 (ATCC: CCL-247), LOVO (ATCC: CCL-229), HT-29 (ATCC: HTB-38), SW480 (ATCC: CCL-228), and melanoma cell line A375 (ATCC: CRL-1619) were purchased from the American Type Culture Collection (ATCC). The human gastric cancer cell line MGC803, human breast cancer cell line MCF-7, and HEK-293T cells were purchased from the Cell Bank of Chinese Academy of Sciences. T2 cell line (TAP-deficient, HLA-A2^+^) was preserved by our institute. It has been proved that LOVO and HCT116 were MSI-H, yet SW480 and HT29 were MSS (29). All the cell lines from ATCC were cultured according to the instructions. MGC803 and HEK293T cells were cultured in Dulbecco’s Modified Eagle Medium (DMEM) (PAN, P04-01550, UK) with 10% fetal bovine serum (Biowest, S1580-500, France). Culture medium (CM) for T lymphocytes was X-VIVO 15 (Lonza, 04-418Q, Switzerland) with 200 IU/ml IL-2 (PeproTech, 200-02, USA), 10 ng/ml IL-7 (PeproTech, 200-07, USA), and 5 ng/ml IL-15 (PeproTech, 200-15, USA).

### Peptides Synthesis

The peptides of NY-ESO-1_157–165_ (SLLMWITQC) and OVA_257-264_ (SIINFEKL) used in this study were synthesized by GL Biochem (Shanghai) Ltd and analyzed to be of > 95% purity by reverse-phase high-performance liquid chromatography as confirmed by mass spectrometry.

### Lentiviral Vector Construction

The codon of α-chain and β-chain gene sequence of NY-ESO-1 specific, HLA-A2-restricted TCR were optimized in reference to the variable region sequence of 1G4 TCR in Robbins’s research, in order to improve the expression level in human cells (25). The Thr-Ser at positions 95-96 of the α chain was mutated to Leu-Tyr to improve its antigen-specific killing function (30). In addition, the mutation of α chain Thr48 and β chain Ser57 into Cys was beneficial to the formation of disulfide bonds and improved the matching success rate of foreign genes (30). The α/β chain genes of TCR were linked by P2A (31), then inserted into the lentiviral vector by double enzyme digestion at BstBI (3521)/EcoRI (5374) sites and verified by sequencing.

Constructed lentiviral vector was co-transfected with assistant vectors (pGP and pVSVG) at the optimal molar ratio of 1:1:1 into HEK-293T cells using the JetPEI (Polyplus Transfection, 101-10N, France) and Optimal medium (Gibco, 31985-070, USA). After 6-8 h, the media was replaced with fresh DMEM with 10% FBS. Then, half of the supernatants were collected after 48 h and fresh media was supplemented to resume incubation. After 72 h, all the supernatants were collected and cell debris scavenged by centrifugation and filtration with 0.45 μm filter. All the viral supernatants were stored at -80° C in equal volumes for transduction after concentration using Beckman Coulter, Optima L-80 XP Ultracentrifuge at 32000 g for 1 h at 4°C.

### Transduction With the NY-ESO-1 TCR

T lymphocytes were sorted from the HLA-A2 positive human PBLs and activated for 48 h *via* Dynabeads Human T-Expander CD3/CD28 Kit (11141D, Thermo Fisher, Norway). 1×10^6^/ml T cells were plated in 12-well plates (Corning, USA) and transduced with the lentivirus at a multiplicity of infection (MOI) of 10 in the presence of polybrene (6 μg/ml) by centrifugation at the speed of 1000 g for 1.5 h. After being incubated at 37°C 5% CO_2_ for 24 h, the cells were centrifuged to remove supernatant and resuscitated with fresh complete culture medium without the lentivirus. Then, the cells were expanded at 37°C, 5% CO_2_ for 5 days and split as necessary.

### Flow Cytometry Analysis

The HLA-A2 positive human PBLs were selected by flow cytometry analysis using phycoerythrin (PE)-conjugated anti-HLA-A2 monoclonal antibody (Biolegend, USA). Cultured for 3 days, the lentivirus transduced human PBLs were stained by fluorescein isothiocyanate (FITC)-conjugated anti-CD3 and PE-conjugated anti-TCR Vβ13.1mAb (Biolegend, USA) for analysis by LSR Fortessa flow cytometer (BD Bioscience, USA).

Allophycocyanin (APC)-NY-ESO-1-MHC tetramer (Biolegend, Flex-T HLA-A2:01 Monomer UVX, USA) was used for the flow cytometric analysis of NY-ESO-1-specific T cells according to the manufacturer’s protocol. Briefly, the manufactured APC-Flex-T tetramer was loaded with NY-ESO-1_157–165_ (SLLMWITQC) by the instruction. 1×10^6^ lentivirus transduced T cells were incubated with 2 μL APC-labeled NY-ESO-1-MHC tetramer for 30 minutes at 37℃. Then, the cells were washed with PBS and analyzed by flow cytometry.

### DAC Exposure

DAC was purchased from Janssen Pharmaceutical Ltd., Xi’an, China for research only. The cell lines were untreated as controls or treated for 72 h with DAC at concentrations of 0.1μM, 1μM, and 10μM, respectively. The media were changed three times every day with fresh medium in the presence of DAC. Then, cells were washed twice with PBS, 24 h after the third medium change, and replaced with medium without DAC. On days 1, 3, and 7, cells were collected for total RNA, DNA, and protein detection. In this experiment, day 1 means the first day after changing media without DAC.

### RT-PCR Analysis

Total RNA was isolated from the cell lines following the instruction of the RNA Extraction Kit (FastGen, FG-80250, China). Synthesis of cDNA was conducted with 2 μg total RNA using the RevTrans Kit (Toyobo, FSQ-101, Japan) and oligo (dT) primers. Quantitative RT-PCR analysis was performed using Roche Cyclin 2.0. The forward primer for NY-ESO-1 was 5’-TGCAGACCACCGCCAACT-3’, and the reverse primer 5’- TCCACATCAACAGGGAAAGCT-3’. For reference gene GAPDH, the forward primers were 5’-GAAGGTGAAGGTCGGAGTC-3’ and the reverse were 5’-GAAGATGGTGATGGGATTTC-3’. Samples were normalized by dividing the copy number of the NY-ESO-1 gene by that of GAPDH.

### Western Blot Assay

Proteins were extracted from the control and the DAC-treated cells and quantified using the BCA protein Assay Kit (Thermo, 23225, USA). Western blot was performed as previously described (32). NY-ESO-1 protein was detected using anti-NY-ESO-1 mAb (Invitrogen, 35-6200, clone: E978, USA) at 1:200 dilution and GAPDH reference protein using anti-GAPDH mAb (Cell Signaling Technology, 2118, clone: 14C10, USA) at 1:4000 dilution.

### IFN-γ ELISPOT Assay

For IFN-γ production assay by ELISPOT kit (MabTech, 3420-2APT-2, USA), untreated T cells, and T cells transduced with mock or TCR gene (ctrl-T or TCR-T) were used as effector cells (E). To confirm *in vitro* IFN-γ production of the NY-ESO-1 specific TCR-T cells, T2 cells (a B-cell × T-cell hybrid line which carries the HLA-A2 allele but lacks both TAP1 and TAP2 genes) were pulsed with NY-ESO-1_157–165_ peptides (10 ng/ml, 50 ng/ml, or 100 ng/ml, respectively) or irrelevant OVA_257-264_ peptides in medium with 25 μg/ml Mitomycin (Sigma) for 1 h at 37°C, washed thrice, and used as stimulator cells (S). Several tumor cells, including LOVO (HLA-A2^-^/NY-ESO-1^-^), MCF-7 (HLA-A2^+^/NY-ESO-1^-^), MGC803 (HLA-A2^-^/NY-ESO-1^+^), and A375 (HLA-A2^+^/NY-ESO-1^++^) were also used as the stimulator cells in this experiment. 1×10^4^ stimulator cells and 2×10^5^ effector cells were incubated in 0.1 ml culture volume into 96-well polyvinylidene difluoride–backed microplates coated with anti-human IFN-γ mAb.

For exploring *in vitro* IFN-γ production of the NY-ESO-1 specific TCR-T cells combined with DAC, as described in *DAC exposure* above, 0.1 or 1 μM DAC pre-treated SW480 cells were gathered 24 h after the third medium change and used as stimulator cells with normal medium without DAC. The untreated SW480 and A375 cell line were used as controls. 1×10^4^ stimulator cells and effector cells at series ratios (E/S=10, 20, 40) were incubated in 0.1 ml culture volume into 96-well polyvinylidene difluoride–backed microplates coated with anti-human IFN-γ mAb. After incubation at 37°C for 24 h, cells were removed and the plates were processed following the manufacturer’s procedures. Resulting spots were counted using CTL-ImmunoSpot S6 Ultimate Micro Analyzer (CTL, Cleveland, USA).

### Cytotoxicity Assays

Cytotoxicity assays were done using a standard LDH assay (Promega, G1780; USA). The tumor cells, as stimulators used in ELISPOT assay as described above, were target cells (T) in this LDH assay. T cells transduced with mock or TCR genes were used as effector cells (E). Shortly, 1×10^4^ target cells and effector cells at series ratios (E/T=10, 20, 40) were incubated in a 100 μL culture volume in 96-well round-bottomed plates and cocultured at 37°C for 4 h. The supernatant (100 μL) was collected from each well and diluted to detect the LDH release. The luminescence signal was recorded by iMark Microlate Absorbance Reader (Bio-Rad, USA) under the corresponding program. percentage of specific lysis was calculated by the following formula: Specific lysis =100 × (Mean Experimental LDH Release – Mean spontaneous release)/(Mean Maximum LDH Release – Mean spontaneous release). Spontaneous and maximum releases were determined by incubating the target cells with medium alone or 1% Triton X-100, respectively. Spontaneous release was always <15% of maximum release. The SD of quadruplicate wells was <15%.

### Adoptive Cellular Transfer to Mice Models

Animal experiments were performed in compliance with the regulations of the Animal Ethical Committee of the Naval Medical University Animal Care and Use Committee. To confirm *in vivo* the tumor inhibition effects of the NY-ESO-1 specific TCR-T cells, A375 (HLA-A2^+^/NY-ESO-1^++^) cell line was a good candidate for target. The BALB/c nude mice were inoculated subcutaneously with 1×10^7^ A375 cells in the lateral flank area. To explore *in vivo* anti-tumor effects of the NY-ESO-1 specific TCR-T cells combined with DAC, SW480 (HLA-A2^+^/NY-ESO-1^±^), a DAC-sensitive CRC cell line with MSS was selected as a target. As described in *DAC exposure* above, 1 μM DAC pre-treated SW480 cells were gathered 24 h after the third medium changing and adjusted to a concentration of 1×10^8^ cells/ml with normal medium without DAC. 100 μL cell suspension were inoculated subcutaneously in the lateral flank area of the NPG mice. 7-10 days after tumor bearing, mice were injected intravenously with 1×10^6^ per mouse the NY-ESO-1 specific TCR-T cells. This adoptive transfer was done once a week for three times. Mice in control groups received the same amount of mock-transduced human lymphocytes or the same volume of PBS. Tumor sizes were measured by vernier caliper (Sata, China) every 3 days and calculated by the formula: tumor volume= (L×W^2^)/2 (L and W represented the longest and shortest diameters). The survival of the tumor-bearing mice was observed and recorded every 3 days.

### Statistical Analysis

All statistical analyses were performed using GraphPad Prism 7.0 software (InStat, GraphPad Software) and SPSS 21.0 software (IBM Corporation, USA). Statistical significance was determined utilizing two-tailed unpaired Student’s t-tests (for comparison of two groups) and one-way analysis of variance (for multiple group comparisons) with α=0.05. Significance calculated by linear regression for tumor measurements against the untreated. For survival analysis, log-rank tests (Mantel–Cox) were used to compare between-group differences in survival curves. All *P* values reported were two sided and *P*<0.05 was considered as significantly different.

## Results

### Preparation and Verification of NY-ESO-1 Specific TCR-T Cells

**Figure 1:**
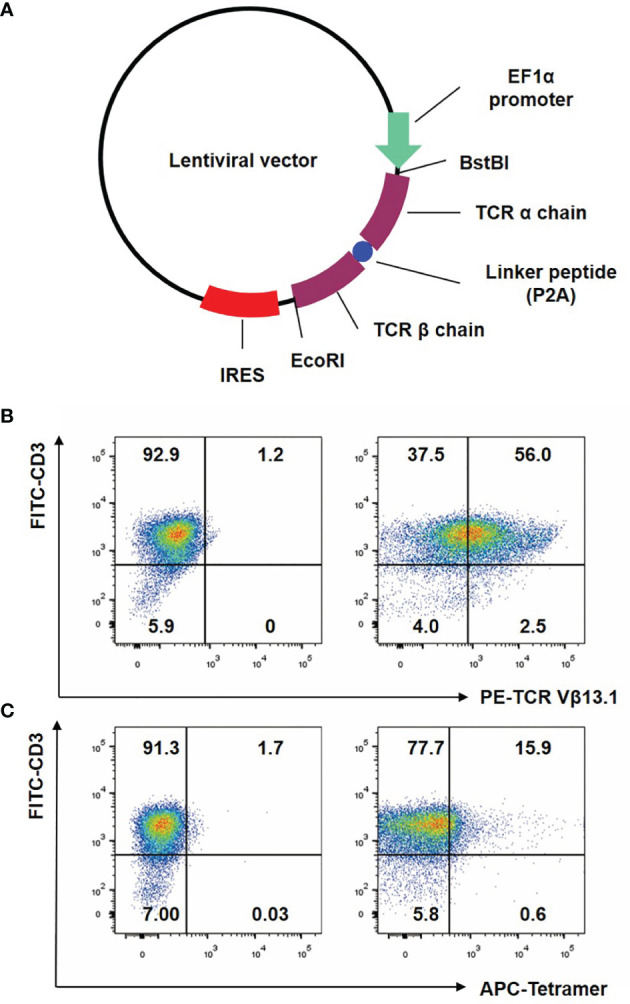
Construction of lentiviral vector and preparation of NY-ESO-1 TCR transduced T lymphocytes. **(A)** The construction of lentiviral vector encoding the NY-ESO-1 TCR gene. **(B)** T cells were analyzed by FACS with FITC-conjugated anti-CD3 antibody and PE-conjugated anti-TCR Vβ13.1 antibody that recognized the β chain of the 1G4-α95:LY. **(C)** T cells were analyzed by FACS with APC-conjugated HLA-A2 tetramer containing the NY-ESO-1_157–165_ (SLLMWITQC). The percentage of cells in each quadrant was indicated. Representative of three independent experiments.

We purposefully constructed the NY-ESO-1 specific TCR (**Figure 1A**), with optimized Thr48 on the α chain and Ser57 on the β chain with cysteines which would form an interchain disulfide bond between the TCR constant regions (30). The bond would improve the matching rate of exogenous TCRs. T cells were analyzed for the transgenic specific TCR Vβ13.1 expression (25) after anti-CD3/CD28 beads stimulation by flow cytometry. We observed that more than half of the transduced T cells expressed the desired transgenic TCR (**Figure 1B**). Tetramer assay was used to detect the binding affinity of the transgenic TCR and NY-ESO-1. NY-ESO-1 specific tetramer was an oligomer form of four MHC class I molecules loaded with NY-ESO-1 peptides, which could bind to the certain TCR on the NY-ESO-1 specific CD8^+^ T cells. We found that 15.9% of the transduced T cells were stained with the tetramer (**Figure 1C**), which indicated that the NY-ESO-1 TCR-T cells were constructed successfully.

### Specific Cytotoxicity of the NY-ESO-1 TCR-T Cells *In Vitro*

**Figure 2:**
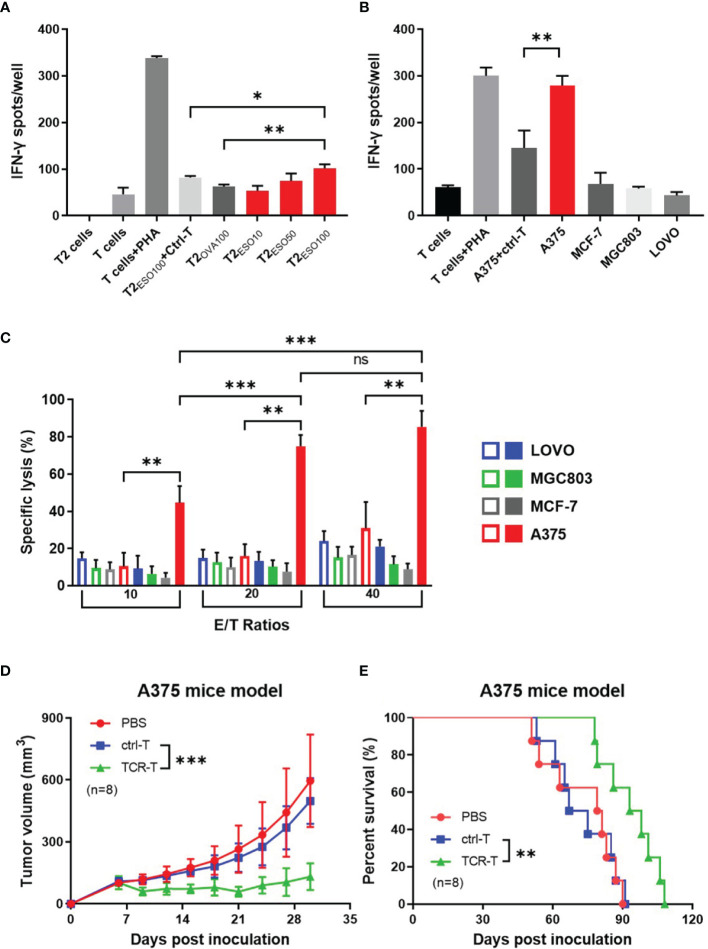
*In vitro and in vivo* specific cytotoxicity of the NY-ESO-1 TCR-T cells. **(A)** IFN-γ ELISPOT assay: T cells transduced with mock gene were used as ctrl-T cells, and the NY-ESO-1 TCR-T cells as effectors. T cells were co-cultured with T2 cells, which were used as stimulators, and loaded with different concentrations of cognate antigens, respectively. The unloaded T2 cells alone and T cells alone served as background controls, while the T cells+PHA served as a positive control. ESO10/50/100 indicated the concentration of antigens at 10 ng/ml, 50 ng/ml, 100 ng/ml NY-ESO-1 peptides (p157-165) respectively, and OVA100 indicated 100 ng/ml irrelevant OVA peptides (p257-264). The effector-stimulator ratio was 20:1 in this experiment. Spots of IFN-γ were counted and analyzed. PHA: Phytohaemagglutinin, a selective T cell mitogen for T cell activation. *P<0.05. **P<0.01. **(B)** IFN-γ ELISPOT assay: ctrl-T cells or the NY-ESO-1 TCR-T cells were co-cultured with tumor cells expressed different genotypes of HLA-A2 and NY-ESO-1, which served as stimulators. The T cells alone and tumor cells alone (not shown) served as background controls, while the T cells+PHA served as a positive control. The effector-stimulator ratio was 20:1 in this experiment. The spots of IFN-γ were counted and analyzed. **(C)** LDH assay: ctrl-T cells (empty column) and the NY-ESO-1 TCR-T cells (full column) were used as effectors and the tumor cells with different genotypes of HLA-A2 and NY-ESO-1 were used as targets. The effectors(E) and targets(T) were co-cultured at different E/T ratios as indicated. Spontaneous release of target cells was less than 20%. Results shown were mean ± SD of quadruplicate wells. ns: no significance. **P<0.01; ***P<0.001. **(D)** Antitumor effects of the TCR-T cells on A375 xenograft nude mice. The PBS, ctrl T cells or the TCR-T cells were infused three times weekly *via* tail vein. The tumor size was measured every three days. Bars, mean ± SD. ***P<0.001. **(E)** Kaplan-Meier survival plot. The survival curve was analyzed by log-rank test. **P= 0.0054.

Firstly, NY-ESO-1 TCR-T cells were co-cultured with T2 cells loaded with NY-ESO-1 derived or irrelevant OVA derived peptides to determine whether the NY-ESO-1 TCR transduced cells could recognize the surface epitope and present cytotoxicity. Antigen-specific IFN-γ release was detected by ELISPOT assay. As shown in the **Figure 2A**, the TCR-T cells could successfully recognize the T2 cells loaded with NY-ESO-1 peptides and release IFN-γ specifically in a concentration-dependent way, but not those loaded with the OVA peptides.

We then used several cancer cell lines with different phenotypes of HLA-A2 or NY-ESO-1 as target cells to determine the specific cytotoxicity of the TCR-T cells *in vitro* by LDH assay (**Figure 2C**) and IFN-γ release by ELISPOT (**Figure 2B**). LDH in the target cells was released in the medium, which was a reliable indicator of cell lysis. As shown in **Figure 2C**, the TCR-T cells exhibited a NY-ESO-1-specific killing against A375 (HLA-A2^+^/NY-ESO-1^++^), but failed to lyse LOVO (HLA-A2^-^/NY-ESO-1^-^), MCF-7 (HLA-A2^+^/NY-ESO-1^-^), and MGC803 (HLA-A2^-^/NY-ESO-1^+^) cells. In comparison, no specific lysis was observed for the mock-transduced T cells. In ELISPOT assay, the target cells in cytotoxicity assay were used as stimulators. As shown in **Figure 2B**, the NY-ESO-1 TCR transduced T cells elicited significant IFN-γ production once received *in vitro* stimulation of A375, and less IFN-γ was produced in LOVO, MCF-7, and MGC803 groups. The mock-transduced T cells did not show significant IFN-γ production on stimulation of the A375 cells we used. Together with the above data, the T cells transduced by NY-ESO-1 TCR were antigen specific and HLA-A2 restrictive.

### Potent Anti-Tumor Effect of the NY-ESO-1 Specific TCR-T Cells *In Vivo*

We subsequently explored the anti-tumor capability of the T cells transduced with NY-ESO-1 TCR *in vivo*. Nude mice were inoculated with A375, a human HLA-A2^+^/NY-ESO-1^++^ melanoma cell line and 7 days later were injected by tail intravenous with the T cells transduced with NY-ESO-1 TCR every three times per week. We found that adoptive transfer of the NY-ESO-1 TCR-T cells was able to significantly inhibit A375 growth in mice (**Figure 2D**) and prolong the survival time (**Figure 2E**); however, no marked tumor growth inhibition or survival improvement was observed in other groups. As mentioned previously, we constructed the NY-ESO-1 specific TCR-T cells successfully to recognize and kill tumor cells by means of antigen specificity and HLA-A2 restriction *in vitro* and *in vivo*.

### Augmented Expression of NY-ESO-1 in the Tumor Cell Lines by DAC Treatment

**Figure 3:**
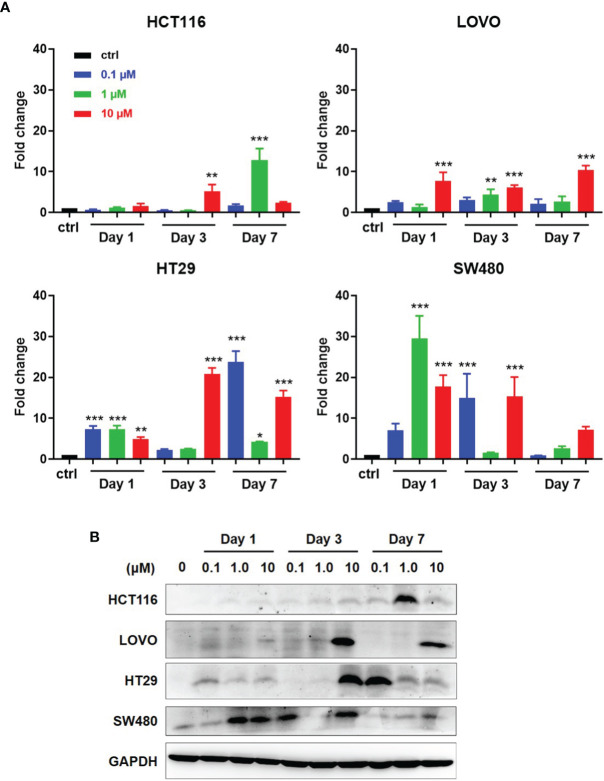
Induction of antigen expression by DAC on CRC cell lines with MSI or MSS. **(A)** After exposed at various lengths of time and at various concentrations of DAC, NY-ESO-1 transcript expression in the indicated CRC cell lines, including LOVO and HCT116 with MSI-H, and SW480 and HT29 with MSS, was assayed by RT-qPCR. The results were normalized to GAPDH and the baseline of untreated groups (controls) was the mean value of one experiment performed in technical triplicate. The results of fold changes were analyzed in comparison to the DAC untreated group as control. *P<0.05, **P<0.01, ***P<0.001. **(B)** NY-ESO-1 protein expression in the above cell lines was detected by Western blot assay. The proteins on the cell membranes were captured by the anti-NY-ESO-1 mAb (E976). GAPDH was used as a loading control.

As reported, DAC can induce and promote the expression of some tumor associated antigens (TAAs), including NY-ESO-1. We first detected the NY-ESO-1 expression in DAC-treated four CRC cell lines, HCT116 and LOVO with MSI, HT29 and SW480 with MSS. Cells were conditioned with DAC for 72 h at different concentrations and harvested in a series of time as indicated in **Figure 3**. Quantitative RT-PCR analysis of NY-ESO-1 mRNA was performed on total RNA extracted from treated cells at days 1, 3, 7 and the untreated cells. We observed that DAC treatment reproducibly elicited induction of NY-ESO-1 gene in the MSS cell lines HT29 and SW480 that increased by 20-30 folds, while 5-10 folds in MSI cell lines LOVO and HCT116. The NY-ESO-1 mRNA expression in HT29 and SW480 cells with MSS was enhanced to a higher expression level than the cell lines with MSI (**Figure 3A**). It also took longer time to induce the expression of NY-ESO-1 in MSI-H cell lines than MSS (**Figure 3A**).

Western blot analysis was conducted to investigate whether NY-ESO-1 mRNA level correlates with protein expression. Among four cell lines with MSS or MSI, NY-ESO-1 expression by LOVO, HT29, HCT116 was not detected before DAC treatment but significantly increased at certain times after *DAC exposure* above (**Figure 3B**). The NY-ESO-1 protein level in MSS cell line SW480 was remarkably up-regulated after DAC treatment (**Figure 3B**). Time course experiments revealed similar kinetics compared to that shown by qPCR. Data showed that DAC could change the TAA expression in MSS CRC cell lines, which suggested the enhanced immunogenicity of the tumor cells.

### More Efficient Specific Lysis to DAC-Treated Tumor Cells by the TCR-T Cells

**Figure 4:**
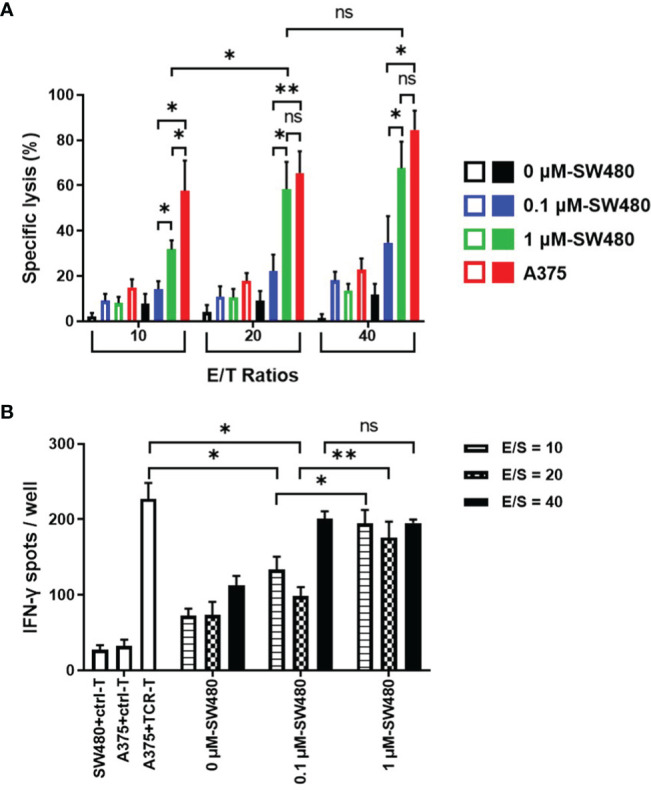
More potent specific lysis to the DAC treated tumor cell lines by the TCR-T cells *in vitro*. **(A)** LDH assay for the cytotoxicity. The TCR-T cells were used as effector cells (E) and SW480 cells treated without or with 0.1 μM, 1 μM DAC as target cells (T). Various E/T ratios were tested as indicated. LDH spontaneous release of the target cells was less than 20%. Results shown were mean ± SD of quadruplicate wells. **(B)** ELISPOT assay for IFN-γ release by the TCR-T cells. The target cells in the cytotoxicity assay were used as stimulators (S). SW480 cells were treated without or with 0.1 μM, 1 μM DAC. Various E/S ratios were tested as indicated. A375 melanoma cell line was selected to be the positive stimulator cells for the high-expressed NY-ESO-1. The IFN-γ spot-forming cells (SFCs) were counted and analyzed by CTL-ImmunoSpot S6 Ultimate Micro Analyzer. ns: no significance. *P<0.05; **P<0.01.

To explore the therapeutic efficacy of the TCR-T cells combined with DAC *in vitro*, DAC-treated SW480 cells were used as the target cells in cytotoxicity assay and stimulator cells in the IFN-γ ELISPOT assay. HLA-A2 positive melanoma cell line A375 with highly expressed NY-ESO-1 was served as a positive control. Cytotoxicity assays were done using a standard LDH-release assay (**Figure 4A**). The TCR-T cells exhibited a NY-ESO-1-specific killing against 1 μM DAC-treated SW480 cells at all three ratios of effectors(E) and the target(T) cells. In comparison, no specific lysis was observed to the untreated SW480 cells. Lysis of SW480 cells treated with 0.1 μM DAC were observed at the 40:1 ratio of E/T cells but not at the 10:1 and 20:1 ratio. Furthermore, the cytotoxicity of the TCR-T cells against target cells treated by 1 μM DAC was more potent than those treated by 0.1 μM DAC. The results indicated that these TCR-T cells were NY-ESO-1 specific and the cytotoxic effects might be associated with the DAC concentration and the ratio of E/T cells.

As shown in the **Figure 4B**, being the stimulator cells in IFN-γ ELISPOT assay, both 0.1 μM and 1μM DAC treated SW480 cells could specifically elicit IFN-γ production by the NY-ESO-1 TCR-T cells, more significantly than those without DAC treatment, regardless of the ratio of the effector (E) and the stimulator (S) cells. Moreover, at the 10:1 and 20:1 ratio of the E/S cells, more IFN-γ-secreting TCR-T cells were counted in 1 μM DAC treatment group than 0.1 μM DAC group.

It was noteworthy that no significant difference was observed between the cytotoxicity efficiency of the TCR-T cells to 1 μM DAC treated SW480 cells (E/T ratios 20:1 and 40:1) and the A375 positive control target cells, which was confirmed to be highly expressive of NY-ESO-1 and HLA-A2. The findings were additionally confirmed by measuring IFN-γ secretion from the TCR-T cells in contact with 1 μM DAC treated SW480 cells. Together, exposure to DAC could improve the expression of NY-ESO-1 which increased immunogenicity of the tumor cells and the sensitivity to the TCR-T cells. The results suggested that 1 μM should be a candidate dose for DAC application in follow-up *in vivo* experiments, even in clinical trials.

### More Potent Anti-Tumor Effect of DAC and the TCR-T Cells Synergistic Therapy in SW480 NPG Mice

**Figure 5:**
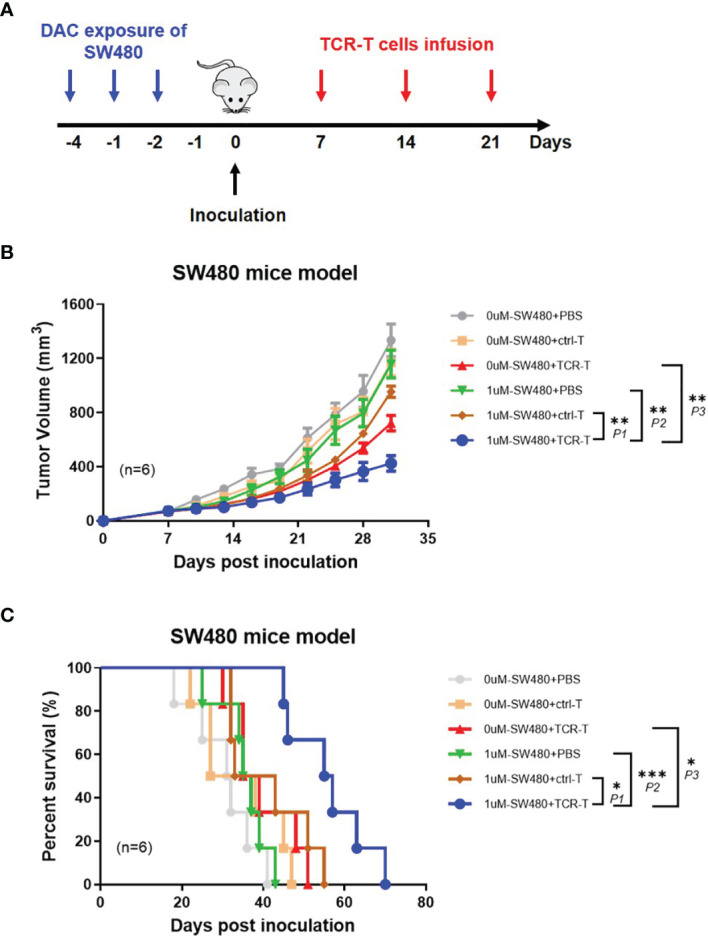
Adoptively transferred TCR-T cells more remarkably inhibit the growth of DAC pre-treated tumor expressing NY-ESO-1 and HLA-A2 *in vivo*. **(A)** The schematic layout. NPG mice (NOD-Prkdc^scid^Il2rg^null^) were inoculated with DAC treated or untreated SW480 cells, and then transferred with PBS, the ctrl-T cells or the TCR-T cells as indicated in Materials and Methods. **(B)** Tumor growth curves. Tumor growth was monitored by measuring the diameter of the tumor every 3 days and recorded as the tumor volume by formula: V= (L×W^2^)/2 (L and W represented the longest and shortest diameters). Bars, mean ± SD. *P1 =* 0.0032; *P2 =* 0.0014; *P3 =* 0.0073. **(C)** Survival rate of the tumor bearing mice. The survival curve was analyzed by log-rank test. *P1 =* 0.0243; *P2 =* 0.0005; *P3 =* 0.0142.

In order to demonstrate the anti-tumor effects of the combined DAC and TCR-T cells *in vivo*, NPG mice were inoculated, subcutaneously, on the lateral flanks with 1 μM DAC treated or untreated SW480, a human HLA-A2^+^/NY-ESO-1^±^ colorectal cancer cell line with MSS and 7 days later injected by tail intravenous with the TCR-T cells three times every week (**Figure 5A**). We found, with excitement, that the TCR-T cells were able to significantly inhibit DAC treated SW480 growth in NPG mice while no marked tumor growth inhibition or survival improvement was observed in all other groups (**Figure 5B**). All the mice developed palpable tumors 6 days after tumor inoculation, whereas the tumor growth inhibition was significant in mice inoculated with DAC treated SW480 cells and received adoptive transfer of the TCR-T cells. All mice in the PBS control groups died between day 18 and 43 after tumor inoculation. In contrast, the death was not observed until day 45 after DAC-treated SW480 tumor inoculation and the TCR-T cells treatment, and in this group, 80% of the mice survived for more than 50 days after the tumor inoculation (**Figure 5C**). A weakly protective capacity of the TCR-T cells also occurred in mice inoculated with untreated SW480 cells and that received the TCR-T cells adoptive transfer. However, tumors were not completely rejected. We failed to observe any sign of tumor growth inhibition or improvement in survival in tumor-bearing mice receiving the control T cells, which showed no significant difference in tumor growth from the PBS treatment control group. The data above showed that the TCR-T cells could result in a more potent protective immune response against DAC pre-treated HLA-A2^+^/NY-ESO-1^+^ tumor cells than those without DAC pretreatment, convincingly suggesting that the synergistic therapeutic strategy of DAC and the antigen specific TCR-T cells was an effective immunotherapeutic approach for CRC with MSS.

## Discussion

CRC is the third most common cancer and a major cause of cancer death worldwide. Only 10-20% of CRC patients are candidates for surgery while others remain palliative intended for only non-surgical treatments (33). In 2017, it was considered hopeful to CRC patients that the FDA approved pembrolizumab, a programmed death 1 (PD-1) inhibitor to treat adult and pediatric patients with unresectable or metastatic, MSI-H or dMMR solid tumors, regardless of tumor site or histology (34, 35). However, CRC is categorized into tumors where only 15% are dMMR/MSI-H, and 85% are MSI-L or MMS and are largely unresponsive to current immune checkpoint inhibitors (36, 37). Thus, for MSI-L or MSS CRC patients, there is an urgent unmet need to develop more effective treatments (38). TCR-engineered T (TCR-T) cells, one of the most important autologous cell therapies, represent the fastest growing immuno-oncology sector and is at the forefront of the next generation immuno-therapeutic approaches (39–41). Compared to chimeric antigen receptor T (CAR-T) cells, TCR-T cells can recognize the entire repertoire of target epitopes from proteins residing within any subcellular compartment, including membrane, cytoplasm, and nucleus which offers a broader spectrum of targets and possible utilities (42). Meanwhile, TCR-T cells use physiological CD3 complex to transduce activation signals which make the response to epitope, whose densities are many folds smaller than required for CAR-signaling (43–46).

In this study, we successfully constructed the NY-ESO-1 specific optimized TCR-T cells which exhibited an antigen-specific killing against the target cells expressed NY-ESO-1 in an HLA-A2 restrictive manner *in vitro* and *in vivo*. Moreover, the cytotoxic effects of the TCR-T cells were related to the amount of NY-ESO-1 expressed by the target cells, reflected in the specific secretion of IFN-γ from the TCR-T cells by antigen-loaded T2 cells stimulations in a concentration-dependent way. NY-ESO-1 is a member of the cancer-testis antigens (CTAs) family, which is frequently expressed in diverse epithelial malignancies, such as melanoma, colorectal cancer, breast cancer, and bladder cancer (47, 48). NY-ESO-1 is regarded as an ideal immune target of cancer due to its exquisitely specific expression of restriction in normal tissues but a fairly widespread occurrence in cancer (49–52). NY-ESO-1 specific TCR-engineered T cells have generated clinical responses in patients with advanced multiple myeloma, synovial cell sarcoma, and melanoma. The product developed by Adaptimmune has been approved by the FDA as an orphan drug for sarcoma. Herein, we constructed the NY-ESO-1 specific TCR-T cells successfully and testified that in A375 tumor bearing mice, adoptive transfer of the NY-ESO-1 TCR-T was able to significantly inhibit A375 growth and prolonged the survival time. However, no marked tumor growth inhibition or survival improvement was observed in PBS or control T cells group.

Actually, SW480 (HLA-A2^+^/NY-ESO-1^±^), a typical CRC cell line with MSS, failed to be lysed by the TCR-T cells regardless of the E/T ratio (**Figure 4A**), which resulted from the poor expression of NY-ESO-1 (**Figure 3**). Simultaneously in the ELISPOT assay, the NY-ESO-1 TCR transduced T cells elicited less IFN-γ production once received *in vitro* stimulation of SW480 than of A375 (**Figure 4B**). These results emphasized that antigen is the first and critical step to determine the effectiveness of TCR engineered T cells. Furthermore, to improve the certain antigen expression of the MSS CRC may be a breakthrough to increase sensitivity to the TCR-T cell therapy (53).

Decitabine (DAC) is one of the demethylation agents which has been reported to induce and improve several TAAs, such as MAGE-A1, MAGE-A2, MAGE-A3 and MAGE-A6 (54–56), and especially induce and/or upregulate the expression of NY-ESO-1 in melanoma (30) and renal cancer cells but not in normal epithelial cells (57). We confirmed that DAC treatment could reproducibly induce NY-ESO-1 gene in the MSS cell lines HT29 and SW480 which was several times that of the MSI-H cell lines LOVO and HCT116. The coherent result was observed in the NY-ESO-1 protein expression in above-mentioned four CRC cell lines, especially in SW480 cells (**Figure 3B**). By genome-wide methylation sequencing, we found that DAC could decrease the methylation level of NY-ESO-1 promoter region, resulting in reactivation and expression of the NY-ESO-1 gene (data not shown). Since the poor immunogenicity is one of the most prominent drawbacks of the CRC with MSS for immunotherapy, DAC may reverse it by improving certain antigen expressions.

Subsequently, we combined the NY-ESO-1 TCR-T cells with DAC to explore the symphysial efficacy to the CRC with MSS. In cytotoxicity assays, the TCR-T cells showed no lysis to the untreated SW480 cells, however, displayed a significant NY-ESO-1-specific killing of 1 μM DAC-treated SW480 cells at all E/T ratios. Lysis of SW480 cells treated with 0.1 μM DAC was observed only at 40:1 E/T ratio which was much less than the target cells treated by 1 μM DAC. Moreover, HLA-A2 positive melanoma cell line A375 served as a positive control because of its highly expressed NY-ESO-1. No significant difference was observed between the cytotoxicity efficiency of the TCR-T cells to 1 μM DAC treated SW480 cells (E/T ratios 20:1 and 40:1) and the A375 cells. These findings were further confirmed by measuring IFN-γ secretion from the TCR-T cells in contact with stimulator cells which were used as the target cells in cytotoxicity assay. In tumor bearing NPG mice, the TCR-T cells were able to significantly inhibit DAC-treated SW480 growth while no marked tumor growth inhibition or survival improvement was observed in all other groups. Consequentially, the potent combined effects of DAC in antigen promotion and the TCR-T cells in cytotoxicity enhancement were clearly shown in the above results, which strongly suggested that the synergistic strategy of DAC and the antigen specific TCR-T cells was a potential immunotherapeutic approach for CRC with MSS in clinic. Furthermore, DAC, or certain demethylation agents, followed by TCR-T cells or other cellular immunotherapy, could also be extended to the treatment of a variety of malignant tumors.

To date, cancer therapies have started to move towards more complex approaches that incorporate multiple therapeutic strategies (58–62). We conclude by discussing how TCR-T cell-based immunotherapies will achieve broader dissemination through combination with DAC, which may mediate regression of solid tumors, including immune-checkpoint inhibitor refractory cancers. Moreover, recognition of TCR to epitopes derived from intracellular proteins or cell surface origin enables TCRs to detect a broader universe of targets, such as neoantigens, cancer germline antigens, and viral oncoproteins. And DAC-based synergistic strategy can be applied to a wider range of solid tumors, such as liver cancer and gastric cancer.

## Data Availability Statement

The original contributions presented in the study are included in the article/supplementary material. Further inquiries can be directed to the corresponding authors.

## Ethics Statement

The animal study was reviewed and approved by Animal Ethical Committee of the Naval Medical University Animal Care and Use Committee.

## Author Contributions

Conception and Design, YW and TW; Acquisition of Data, GY, WW, JX, and XH; Analysis and Interpretation of Data, GY, WW, XH, JX, and RX; Drafting the manuscript, GY and YW; Final approval of the completed manuscript, YW and TW. All authors read and approved the final manuscript.

## Funding

This work was supported by the Grants from National Key R&D Program of China (Nos. 2016YFC1303504), the National Natural Science Foundation of China (Nos. 81901686, 81671644, 82071796, 81471625) and biomedical project of “Science and Technology Innovation Action Plan” of Shanghai (Nos. 19431901500).

## Conflict of Interest

The authors declare that the research was conducted in the absence of any commercial or financial relationships that could be construed as a potential conflict of interest.

## Publisher’s Note

All claims expressed in this article are solely those of the authors and do not necessarily represent those of their affiliated organizations, or those of the publisher, the editors and the reviewers. Any product that may be evaluated in this article, or claim that may be made by its manufacturer, is not guaranteed or endorsed by the publisher.
